# Factors associated with nurses' user resistance to change of electronic health record systems

**DOI:** 10.1186/s12911-021-01581-z

**Published:** 2021-07-17

**Authors:** Younghee Cho, Mihui Kim, Mona Choi

**Affiliations:** 1 College of Nursing, Mo-Im Kim Nursing Research Institute, Yonsei University, 50-1 Yonsei-ro, Seodaemun-gu, Seoul, 03722 Republic of Korea; 2 Department of Digital Health, Samsung SDS, Seoul, Republic of Korea; 3 College of Nursing, Brain Korea 21 FOUR Project, Yonsei University, Seoul, Republic of Korea

**Keywords:** User resistance behavior, Resistance to change, Electronic Health Record system

## Abstract

**Background:**

Electronic health record (EHR) systems often face user resistance in hospitals, which results in a failure to acquire their full benefits. To implement the EHR successfully, it is crucial to reduce nurses’ resistance to use the system. This study aimed to investigate the factors associated with nurses’ resistance to use the EHR system.

**Methods:**

A descriptive correlational study was conducted with nurses working at four university hospitals in Korea using self-administered questionnaires to measure user resistance behavior, resistance to change, perceived usefulness, perceived ease of use, perceived value, colleagues’ opinions, self-efficacy for change, and organizational support for change. Path analysis was performed to examine direct and indirect association with user resistance behavior.

**Results:**

A total of 223 nurses completed the questionnaires. All seven factors were found to be significantly associated with user resistance, either directly or indirectly. The total effect on user resistance behavior was highest in *resistance to change* (0.65), followed by *perceived usefulness* (− 0.33); both had direct but no indirect effects. Conversely, *self-efficacy for change* (− 0.25), *perceived value* (− 0.21), *colleagues’ opinions* (− 0.16), *perceived ease of use* (− 0.16), and *organizational support for change* (− 0.05) had indirect but no direct effects.

**Conclusions:**

The study examined the factors associated with nurses’ user resistance behavior after the implementation of a new EHR system. These findings could help hospitals develop better EHR implementation strategies to reduce user resistance behavior among the nursing staff.

**Supplementary Information:**

The online version contains supplementary material available at 10.1186/s12911-021-01581-z.

## Background

In recent decades, Electronic Health Record (EHR) systems have developed remarkably. Between 2010–2015 in Korea, their adoption rate increased from 50.2 to 97.3% in tertiary teaching hospitals and from 35 to 91.4% in general hospitals [1]. In addition, the quality of EHR has improved; the comprehensive level of EHR increased from 13.2 to 17% in tertiary teaching hospitals and from 3.6 to 10.8% in general hospitals [1]. The hospitals that adopted all functions, including seven types of electronic clinical documentation, six result views, five Computerized Physician Order Entry (CPOE) systems, and six decisional supports in all clinical units were classified as having a comprehensive level of EHR [2]. EHR systems are reducing medical errors, costs, and patients’ waiting time before receiving assistance, providing a more convenient system to archive patient data, and improving access to patient information and efficiency [1, 3–7]. These result in an overall improvement in quality of care [3] and patient safety [4–7].

The implementation of information systems has brought about acceptance as well as resistance [8, 9]. Some studies have indicated that physicians’ user resistance is the primary barrier in adopting information systems in hospital settings [6, 10, 11]. Kirkley and Stein [12] indicated that resistance among nurses also plays a key part in the implementation of new nursing information systems. According to Kim and Kankanhalli [13], user resistance to information systems has proven to be the biggest challenge in implementing large-scale systems.

Resistance to information systems is not restricted to the refusal to use the system. To understand user resistance, it is necessary to understand user acceptance as well because they should not be regarded as mere opposites [14]. Acceptance of information systems does not imply that there is no user resistance, especially in the case of mandatory systems. Acceptance or non-acceptance occurs when there is a voluntary system for personal purposes, such as using email or social networks; however, user resistance tends to manifest when there is a mandatory system [6], such as EHR as well as the large-scale systems that influence the entire hospital workflow [6, 15].

Moreover, acceptance targets a specific information system, while resistance is the general objection to a situation engendering changes by information systems [7]. Therefore, resistance does not focus on a specific information system but on the changes in status quo that are generated by the changes in information systems [7, 16].

Most prior studies regarding the successful implementation of the EHR have focused on user acceptance after the Technology Acceptance Model (TAM) was introduced by Davis [17], and only a few have focused on user resistance, especially quantitative empirical studies [3, 7, 8, 13, 18]. Furthermore, studies involving healthcare providers, especially nurses, in this regard are scarce. Previous studies have shown that nurses can exhibit resistance to information systems, focusing primarily on technical factors, and underestimating the influence of managerial, social, cultural, and behavioral factors [3, 7, 19, 20]. Notably, nurses perform central roles in the healthcare delivery system and comprise the majority of healthcare workers. Therefore, their resistance to new information systems should be considered as a key indicator of how much implementation of the EHR is successful [21, 22].

Thus, it is necessary to understand why users resist new EHR systems while focusing on user resistance behavior in a hospital setting. While there is limited agreement regarding a theoretical model of user resistance behavior, a few prior studies have proposed models of user resistance. Hence, this study aimed to investigate the factors associated with nurses’ user resistance behavior to the EHR. For this purpose, first, we explained the research model of user resistance behavior from prior user resistance and acceptance literature. Second, we empirically tested the research model in a hospital setting. The study could contribute to the development of more adequate strategies to reduce resistance to the EHR based on the theoretical understanding of user resistance behavior.

## Methods

### Theoretical background and hypotheses of research model

**Fig. 1:**
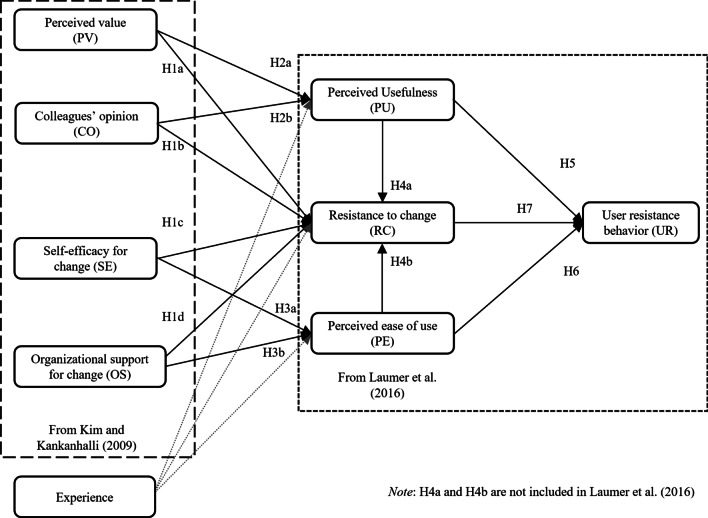
User resistance behavior research model

This study proposes a research model to understand user resistance behavior and its antecedents (Fig. 1). This research model is derived from the user resistance model of Kim and Kankanhalli [13], which explains the factors influencing user resistance behavior based on the status quo bias theory by Samuelson and Zeckhauser [23]. Additionally, this model includes concepts about an individual’s perception that mediate the associated factors and user resistance behavior [7]. These concepts are *resistance to change* (RC), *perceived usefulness* (PU), and *perceived ease of use* (PE) from Laumer et al. [14].

Four factors are included as antecedents of user resistance: *perceived value* (PV), *colleagues’ opinions* (CO), *self-efficacy for change* (SE), and *organizational support for change* (OS). These are derived from the user resistance model of Kim and Kankanhalli [13]. Additionally, the status quo bias theory of Samuelson and Zeckhauser posits that individuals are likely to remain in their current situation; thus, user resistance manifests as a preference to continue using the current system or as a refusal to use a new system [13]. Perceived value is defined by Kim and Kankanhalli as the perceived benefits relative to the costs of implementing a new EHR system [13]. If individuals perceive the change as valuable rather than costly, they are less likely to resist the change. Thus, resistance to change is low if the perceived value of the new EHR is high.

#### H1a:

Perceived value has a negative effect on resistance to change.

The theory of planned behavior (TPB) postulates that social norms affect individuals’ beliefs and behavior [24]; thus, social norms could reinforce or diminish individuals’ status quo bias [13]. In particular, colleagues’ opinions are influential during the implementation of a new EHR system [13]. Thus, resistance to the implementation of a new EHR system would be lower if colleagues’ perceptions of the new system are favorable.

#### H1b:

Colleagues’ opinions have a negative effect on resistance to change.

Generally, individuals dislike losing control because of changes in their work routine, as in the case of implementing a new information system, which reinforces status quo bias [23]; this corresponds to perceived behavioral control of the TPB. Meanwhile, self-efficacy is the internal mean method of perceived behavioral control [25]. According to Kim and Kankanhalli [13], self-efficacy is defined as individuals’ confidence in their ability to adapt to new situations*.* Specifically, a change in the system, such as EHR, causes significant changes to healthcare providers’ working routines, indicating loss of control. Thus, individuals with high self-efficacy are less likely to be awed and more receptive to situations that allow changes in their work. However, those with low self-efficacy are more likely to maintain the status quo and resist change.

#### H1c:

Self-efficacy for change has a negative effect on resistance to change.

According to Kim and Kankanhalli, organizational support, which is included in the external mean method of the status quo’s bias control, is defined as the organization’s perceived facilitation to allow users to adapt to information system-related changes easily [13]. Organizational support encompasses the training of employees in the new system’s processes, guidelines for applying the new way of work, and additional human resources required for the transition period. Consequently, if sufficient organizational support is provided, resistance to change could be reduced.

#### H1d:

Organizational support for change has a negative effect on resistance to change.

The relationships among the four antecedents as well as perceived usefulness or perceived ease of use are derived from the Extended Technology Acceptance Model (TAM2) [26]. In this model, perceived usefulness is deemed high when individuals recognize that new information systems can be useful in terms of job relevance, output quality, and result demonstrability—that is, if individuals believe that a new EHR system is valuable to their work, they would perceive it as useful. Moreover, the TAM2 posits that subjective norms have a positive effect on perceived usefulness and that if co-workers suggest that a system might be useful, their colleagues might come to believe this too [27].

#### H2a:

Perceived value has a positive effect on perceived usefulness.

#### H2b:

Colleagues’ opinions have a positive effect on perceived usefulness.

The Technology Acceptance Model 3 (TAM3) [28] was developed to understand the external variables affecting perceived ease of use; it explains control as a precursor to perceived ease of use, based on the models established by the theory of reasoned action and the TPB [27]. Self-efficacy for change and organizational support (determinants of perceived ease of use) correspond to computer self-efficacy and perception of external control, respectively, in the TAM3. According to the TAM3, individuals with high self-efficacy and those supported by their organization tend to have high perceived ease of use.

#### H3a:

Self-efficacy for change has a positive effect on perceived ease of use.

#### H3b:

Organizational support for change has a positive effect on perceived ease of use.

Enablers and inhibitors should be examined simultaneously, as inhibitors are not the opposite of enablers—they often have been observed to coexist [7]. Four previous studies have included all three factors—perceived usefulness, perceived ease of use, and resistance to change—in their research model to explain intention to use [7, 29, 30] and user resistance behavior [14]. Among these four, three studies show that resistance to change affected perceived usefulness or perceived ease of use, while showing the mediating effect of resistance to change on intention to use [7, 29, 30]. However, the fourth study did not examine the relationship between the three factors [14]. Our study tackles the relationship between all three factors (i.e., perceived ease of use, perceived usefulness, and resistance to change), while treating user resistance behavior as the outcome variable. In other words, we hypothesize that individuals who have higher perceived usefulness or perceived ease of use would have less resistance to change.

#### H4a:

Perceived usefulness has a negative effect on resistance to change.

#### H4b:

Perceived ease of use has a negative effect on resistance to change.

In this study, user resistance in the model was called “user resistance behavior” as explained by Laumer et al. [14]. Both perceived usefulness and perceived ease of use act as essential cognitive determinants to improve acceptance in the TAM and the Unified Theory of Acceptance and Use of Technology [31]. However, these are inhibiting factors regarding user resistance. If individuals feel that new information systems enhance their job performance or require no effort to use, they tend to accept rather than reject the systems.

#### H5:

Perceived usefulness has a negative effect on user resistance behavior.

#### H6:

Perceived ease of use has a negative effect on user resistance behavior.

Moreover, resistance to change is presented by resisting new system based on their perception of status quo bias induced by new information system [7, 13], according to which individuals prefer to maintain their status and avoid changes like the implementation of a new EHR. Individuals with high resistance to change are more likely to present resistance behavior than those with low resistance to change [14].

#### H7:

Resistance to change has a positive effect on user resistance behavior.

### Study setting and participants

This study was a descriptive correlational study conducted in four university hospitals in South Korea that had implemented a new EHR system in the preceding two years. Participants included nurses who worked for at least six months before the change of EHR to ensure familiarity with both the old and new versions of the EHR system. Data were collected between May and July 2020. A total of 240 survey questionnaires were distributed (120, 50, 40, and 30 in each hospital), 235 questionnaires (115, 50, 40, and 30 from each hospital) were collected, and 223 questionnaires were completed. This study was approved by the Institutional Review Board (IRB) of the Yonsei University Health System (IRB number: Y-2019-0193).

### Measurement

The questionnaire consisted of general characteristics and survey items (including user resistance behavior and impact factors). General characteristics included age, sex, job position, period of service, department of service, and period of EHR use. Survey items (34 items) included user resistance behavior (5 items), resistance to change (4 items), perceived usefulness (4 items), perceived ease of use (5 items), perceived value (4 items), colleagues’ opinions (3 items), self-efficacy for change (4 items), and organizational support for change (5 item) (Additional file 1). Those survey items were rated on a 7-point Likert scale ranging from 1 (*strongly disagree*) to 7 (*strongly agree*). The survey items, translated into Korean, which have been used for the corporate information system in prior studies [13, 32, 33], were revised by the researchers to suit the hospital environment in this study.

To measure the content validity of the revised measurement items, a group of experts with experience in the field of health informatics was formed, consisting of five professors of nursing colleges and two doctoral students working at the hospitals. The content validity of the questionnaire was measured by the experts to determine whether the concepts and contents were valid using a 4-point Likert scale ranging from 1 (*not valid at all*) to 4 (*very valid*). Of the 34 items, 32 had an item-content validity index (I-CVI) of 0.8 or higher, and the remaining 2 items (Items 5 and 34) had an I-CVI of 0.71 and 0.57, respectively. Subsequently, items were revised after discussion among the research team, based on the opinions of the experts. The final questionnaire was reviewed by a professor of Korean language and literature to ensure that the grammar of the items was appropriate.

To ensure the reliability of revised measurement items, Cronbach’s alpha coefficient was calculated, all of which were greater than 0.748 (Additional file 1). To validate the survey questionnaires, we assessed convergent and discriminant validity. The composite reliability (CR) and the average variance extracted (AVE) for all constructs should exceed 0.7 and 0.5, respectively [34]. In our study, all CRs exceed 0.82 and all AVEs exceed 0.61. The square root of AVE for each construct exceeded the correlations between the construct and other constructs (Table 3) [35]. Thus, convergent and discriminant validity of the instrument were successfully established. To estimate the overall model fit, chi-square value/degrees of freedom, root mean square error of approximation (RMSEA), comparative fit index (CFI), standardized root mean square residual (SRMR), and Tucker Lewis index (TLI) were used. The fit indices reveal a good model fit with chi-square value/degrees of freedom and RMSEA values of 2.12 and 0.071, respectively. Similarly, the CFI value was 0.911, the SRMR value was 0.062, and the TLI value was 0.899. Thus, all values were acceptable [36]. To assess the extent of common method bias, first Harman’s one-factor test was used. Total variance explained by a single factor was shown to be 42.4%, which was less than the 50% cut-off point [37]. Second, all correlation was less than 0.77 (Table 3). Extremely high correlations (r > 0.9) are the evidence of common method bias [38]. Therefore, no common method bias existed in this study.

### Statistical analysis

General characteristics were presented as means with standard deviation (SD) for continuous variables and as numbers and percentages for categorical variables. Pearson’s correlation coefficients were calculated to examine multicollinearity among variables. Path analysis was conducted to examine the direct and indirect effects of other variables on user resistance behavior. Path analysis is a type of multiple linear regression analysis in which the estimated significance of the different variables can be demonstrated; this includes direct or indirect causal relations between variables. The graphical representation of the path diagram showing the relationship between different variables includes user resistance behavior (dependent), perceived usefulness, perceived ease of use, and resistance to change as endogenous variables, as well as perceived value, colleagues’ opinion, self-efficacy, and organizational support as exogenous variables. All data were analyzed using RStudio software (version 1.3.1056, RStudio, PBC).

## Results

### Participants’ characteristics and descriptive results

Participants’ characteristics are shown in Table 1. Among 223 nurses, 81 (36.3%) were under 30 years old and 71 (31.8%) were between 31 and 40 years old. The number of female nurses was 215 (96.4%). Staff nurses were the majority, at 161 (72.2%); supervising nurses were the minority, at 11 (4.9%). There were 83 nurses (37.2%) with less than 5 years of experience, 47 (21.1%) had between 6 and 8 years of experience, while the majority, 93 (41.7%), had over 9 years of experience. Most of the nurses were working in inpatient units (74.4%), 28 (12.6%) were working in intensive care units, and 13 (5.8%) were working at outpatient units when changing EHR system.

**Table 1:** Participants’ characteristics

Variable	Category	*n*	(%)
(*N* = 223)			
Age (years)	≤ 30	81	(36.3)
(Mean: 34.5, *SD*: 8.6)	31–40	71	(31.8)
	41–50	55	(24.7)
	51–60	16	(7.2)
Sex	Female	215	(96.4)
	Male	8	(3.6)
Position	Supervising nurses	11	(4.9)
	Senior staff nurses	51	(22.9)
	Staff nurses	161	(72.2)
Experience (years)	1–5	83	(37.2)
(Mean: 11.2, SD: 8.8)	6–8	47	(21.1)
	9 and above	93	(41.7)
Working unit	Inpatient units	166	(74.4)
	Intensive care units	28	(12.6)
	Outpatient units	13	(5.8)
	Others	16	(7.2)
Hospitals	A	109	(48.9)
	B	44	(19.7)
	C	40	(17.9)
	D	30	(13.5)

*SD* standard deviation

Table 2 presents descriptive results of variables. On the scale of 7, the mean of user resistance behavior (mean = 3.36, SD = 1.13) was lower than that of resistance to change (mean = 4.03, SD = 1.38). Sixty-eight nurses (30.5%) showed resistance to change, however only twenty-one (9.4%) showed user resistance behavior. Perceived ease of use showed the lowest mean (mean = 3.78, SD = 1.16) among 3 mediating variables. The mean of perceived value (mean = 4.59, SD = 1.24) was highest among four antecedent variables followed by organizational support for change (mean = 4.31, SD = 1.09), self-efficacy for change (mean = 4.10, SD = 1.06), and colleagues’ opinions (mean = 3.85, SD = 1.13).

**Table 2:** Descriptive results

Variables	Score			Mean	SD
	1 ~ 3^†^	4^‡^	5 ~ 7^§^		
(*N* = 223)					
User resistance behavior	81 (36.3)	121 (54.3)	21 (9.4)	3.36	1.13
Resistance to change	49 (22.0)	106 (47.5)	68 (30.5)	4.03	1.38
Perceived usefulness	52 (23.3)	116 (52.0)	55 (24.7)	4.04	1.20
Perceived ease of use	70 (31.4)	114 (51.1)	39 (17.5)	3.78	1.16
Perceived value	24 (10.8)	101 (45.3)	98 (43.9)	4.59	1.24
Colleagues’ opinion	64 (28.7)	118 (52.9)	41 (18.4)	3.85	1.13
Self-efficacy to change	39 (17.5)	129 (57.8)	55 (24.7)	4.10	1.06
Organizational support for change	25 (11.2)	122 (54.7)	76 (34.1)	4.31	1.09

Data are expressed as the n (%)

*SD* standard deviation

^†^strongly disagree ~ slightly disagree

^‡^neutral

^§^slightly agree ~ strongly agree

### Path analysis

Table 3 shows the result of correlation analysis. All correlations were significant except between perceived value and self-efficacy. Regarding user resistance behavior, resistance to change had the highest correlation (0.65), and all other variables were significant (− 0.26 – − 0.43). The correlations between perceived usefulness and perceived value, perceived ease of use and colleagues’ opinion, and perceived ease of use and self-efficacy were higher than 0.6 (0.77, 0.60, 0.65, respectively). Moreover, the correlation between perceived usefulness and colleagues’ opinions as well as perceived value and colleagues’ opinions were higher than 0.5 (0.53, 0.54, respectively).

**Table 3:** Correlation matrix

	UR	RC	PU	PE	PV	CO	SE	OS
UR	0.65							
RC	0.65*	0.79						
PU	− 0.41*	− 0.27*	0.93					
PE	− 0.34*	− 0.48*	0.45*	0.86				
PV	− 0.43*	− 0.28*	0.77*	0.40*	0.92			
CO	− 0.43*	− 0.41*	0.53*	0.60*	0.54*	0.78		
SE	− 0.26*	− 0.38*	0.15*	0.65*	0.13	0.35*	0.79	
OS	− 0.30*	− 0.24*	0.41*	0.52*	0.38*	0.49*	0.38*	0.84

Diagonal shows the squared root of AVE of each variable

*CO* colleagues’ opinion, *OS* organizational support for change, *PE* perceived ease of use, *PU* perceived usefulness, *PV* perceived value, *RC* resistance to change, *SE* self-efficacy for change, *UR* user resistance behavior

^*^P < .05

**Fig. 2:**
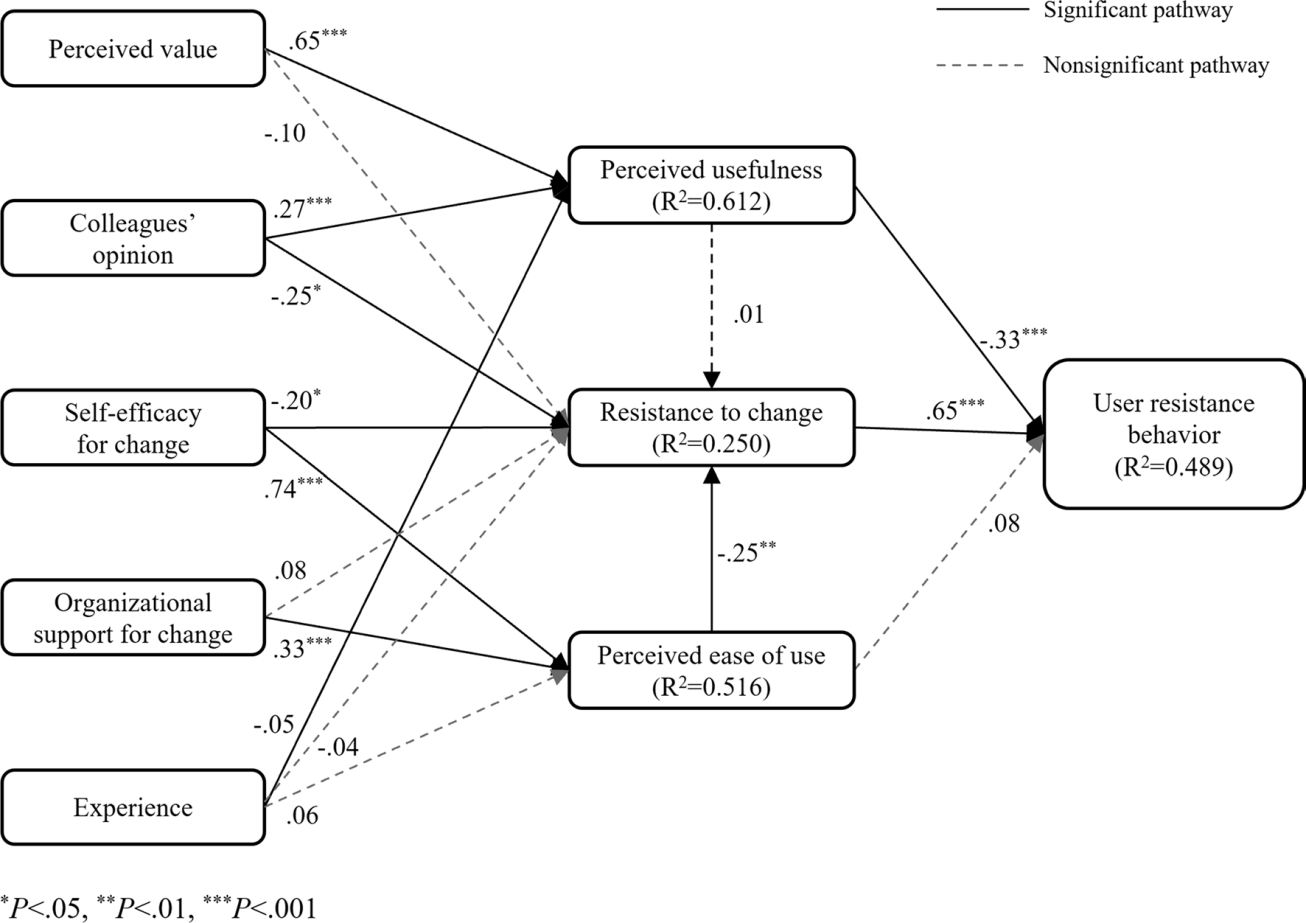
Path analysis results

Figure 2 and Table 4 show the result of path analysis. Colleagues’ opinions (path coefficient =  − 0.25, *P* = 0.043) and self-efficacy (path coefficient =  − 0.20, *P* = 0.037) had a significantly negative impact on resistance to change, while perceived value (path coefficient =  − 0.10, *P* = 0.03) and organizational support (path coefficient = 0.08, *P* = 0.291) were not significant. Both perceived value (path coefficient = 0.65, *P* < 0.001) and colleagues’ opinions (path coefficient = 0.27, *P* < 0.001) had a significantly positive impact on perceived usefulness. Organizational support (path coefficient = 0.33, *P* < 0.001) and self-efficacy (path coefficient = 0.74, *P* < 0.001) were effective on perceived ease of use in a positive manner. Our findings show that the relationship between perceived usefulness (path coefficient = 0.01, *P* = 0.923) and resistance to change was not significant and that the relationship between perceived ease of use (path coefficient =  − 0.25, *P* = 0.001) and resistance to change was significant. Finally, the effect of perceived ease of use (path coefficient = 0.08, *P* = 0.14) on user resistance behavior was not significant; however, perceived usefulness (path coefficient =  − 0.33, *P* < 0.001) had a significantly negative effect on user resistance behavior, and resistance to change (path coefficient = 0.65, *P* < 0.001) had a significantly positive effect on user resistance behavior.

**Table 4:** Path analysis results

Hypotheses		Path coefficient	z statistics	*P* values
H1a	PV → RC	− 0.10	− 1.037	0.30
H1b	CO → RC	− 0.25	− 2.022	0.04*
H1c	SE → RC	− 0.20	− 2.085	0.04*
H1d	OS → RC	0.08	1.056	0.29
	Experience → RC	− 0.04	− 1.022	0.30
H2a	PV → PU	0.65	13.596	< .001***
H2b	CO → PU	0.27	3.740	< .001***
	Experience → PU	− 0.05	− 1.963	0.05
H3a	SE → PE	0.74	10.445	< .001***
H3b	OS → PE	0.33	6.097	< .001***
	Experience → PE	0.06	1.885	0.06
H4a	PU → RC	0.01	0.097	0.92
H4b	PE → RC	− 0.25	− 3.313	0.001**
H5	PU → UR	− 0.33	− 5.712	< .001***
H6	PE → UR	0.08	1.476	0.14
H7	RC → UR	0.65	11.353	< .001***

*CO* colleagues’ opinion, *OS* organizational support for change, *PE* perceived ease of use, *PU* perceived usefulness, *PV* perceived value, *RC* resistance to change, *SE* self-efficacy for change, *UR* user resistance behavior

*P < .05, **P < .01, ***P < .001

As shown in Table 5, resistance to change (0.65) had the highest total effect on user resistance behavior, followed by perceived usefulness (− 0.33), both of which have a direct effect. Self-efficacy for change had the third highest total effect on user resistance behavior (− 0.25), with only indirect effects via resistance to change and perceived ease of use-resistance to change. Moreover, organizational support had a total effect of − 0.05 via perceived ease of use-resistance to change. Perceived value and colleagues’ opinions had a total effect of − 0.21 and − 0.16, respectively, via perceived value. Perceived ease of use had a total effect of − 0.16, with only indirect effect via resistance to change. Additionally, self-efficacy had a direct effect of − 0.20 and an indirect effect of − 0.19 via perceived ease of use and had a total effect of − 0.39 on resistance to change.

**Table 5:** Direct, indirect, and total effect on mediating and outcome variables

Variables		Mediating variables			Outcome variable
		Perceived usefulness	Resistance to change	Perceived ease of use	User resistance behavior
Perceived value	Direct effect	0.65	ns		
	Indirect effect		ns		− 0.21
	Total effect	0.65	ns		− 0.21
Colleagues’ opinions	Direct effect	0.27	− 0.25		
	Indirect effect		ns		− 0.16
	Total effect	0.27	− 0.25		− 0.16
Self-efficacy for change	Direct effect		− 0.20	0.74	
	Indirect effect		− 0.19		− 0.25
	Total effect		− 0.39	0.74	− 0.25
Organizational support	Direct effect		ns	0.33	
for change	Indirect effect		− 0.08		− 0.05
	Total effect		− 0.08	0.33	− 0.05
Perceived usefulness	Direct effect		ns		− 0.33
	Indirect effect				ns
	Total effect		ns		− 0.33
Resistance to change	Direct effect				0.65
	Indirect effect				
	Total effect				0.65
Perceived ease of use	Direct effect		− 0.25		ns
	Indirect effect				− 0.16
	Total effect		− 0.25		− 0.16

*ns* non-significant

## Discussion

This study was conducted to identify factors influencing nurses’ user resistance behavior in hospital settings by building a theoretical model considering both resistance and acceptance. All seven factors (i.e., resistance to change, perceived usefulness, perceived ease of use, perceived value, self-efficacy for change, colleagues’ opinions, and organizational support for change) affected user resistance behavior either directly or indirectly. We will discuss our findings with those of previous studies in terms of theory and practice in this discussion section.

### Implications for theory

This study has several implications for user resistance behavior theory. The literature regarding user resistance behavior is limited, even though several prior researches have provided theoretical models, they are fragmented and non-cumulative [7]. Furthermore, research considering both resistance and acceptance are scarce, even though resistance is not the opposite of acceptance and could occur with acceptance simultaneously. Our study adds evidence to the theoretical model of user resistance behavior considering both resistance and acceptance.

First, our study extends the relationship between user resistance behavior and perception of resistance and acceptance. Resistance to change was found to be the strongest predictor of user resistance behavior among mediating factors followed by perceived usefulness and perceived ease of use. To our knowledge, only a single study has explored the association between user resistance behavior and resistance to change, perceived usefulness, and perceived ease of use, where all three factors had a direct relationship with user resistance behavior, but the said study did not explore the indirect effect of the aforementioned factors on user resistance behavior [14]. Conversely, our study showed that perceived usefulness and resistance to change had a direct effect on user resistance behavior, while perceived ease of use did not; however, perceived ease of use had an indirect effect on user resistance behavior via resistance to change. Second, our study provides more explanation on the effect of acceptance on user resistance behavior. Earlier user acceptance studies, examining perceived ease of use, showed inconsistent findings as to its direct effect on the intention to use [39]; however, perceived ease of use has been noted to have an indirect effect on the intention to use through perceived usefulness [40, 41]. Our study reveals that perceived ease of use does not have a direct effect on user resistance behavior which is inconsistent with Laumer et al.’s result [14]; however, it has an indirect effect via resistance to change although Laumer et al. [14] did not examine an indirect effect. Some studies regarding user resistance and acceptance have argued that organizations usually limit their measures to improve ease of use in the technical aspects [19, 20]. However, it is still a useful factor to consider when seeking to reduce user resistance behavior, as is resistance to change, although perceived ease of use is not the only factor influencing user resistance behavior. In contrast, perceived usefulness is a robust and consistent factor influencing user acceptance across user acceptance literature [39]. Similarly, our study shows that perceived usefulness is crucial to reduce user resistance behavior, which is consistent with the findings of Laumer et al. [14]. Therefore, perceived ease of use and perceived usefulness would be effective in both reducing user resistance behavior and improving user acceptance. Third, this study supports a user resistance behavior model based on status quo bias. The findings of this study are overall consistent with Kim and Kankanhalli’s [13] based on status quo bias. All four antecedents drawn from Kim and Kankanhalli’s study influenced user resistance behavior. Self-efficacy for change was found to be the strongest predictor of user resistance behavior followed by perceived value and colleagues’ opinions; organizational support was the weakest. Perceived value was found to be a significant factor in reducing user resistance behavior by increasing perceived usefulness. Notably, perceived value did not influence resistance to change in this study, which is consistent with earlier research [29]. Colleagues’ opinions indirectly reduce user resistance behavior by improving perceived usefulness and reducing resistance to change. This is consistent with the findings of Chi’s study that show the relationship between colleagues’ opinions and resistance to change negatively [29]. Self-efficacy for change could reduce user resistance behavior by reducing users’ resistance to change and increasing perceived ease of use regarding a new system. Additionally, the relationship between self-efficacy and perceived ease of use also supports TAM3 [28]. Our findings show that enhancing organizational support could reduce resistance to change by improving perceived ease of use of a new system, which would, in turn, reduce user resistance behavior. In addition, except perceived value, colleagues’ opinion, self-efficacy for change, and organizational support among antecedents were effective in reducing resistance to change. Self-efficacy for change was the strongest factor influencing resistance to change. In summary, this study adds empirical finings to theoretical models of user resistance as well as technology acceptance.

### Implications for practice

User resistance behaviors are often ignored when EHR is implemented in a hospital, because overt resistance behavior is not common. Although overt resistance behavior does not appear against a mandatory system in which users do not have any choice whether they use it or do not use it, covert resistance behavior could still occur [19, 20]. Covert resistance behavior might be more dangerous because it causes inappropriate use of EHR systems, which could generate significant risk in a hospital setting [15]. Hospital management could utilize the findings of this study to implement EHR successfully by reducing user resistance behavior in hospitals.

Nurses were observed to manifest less resistance behavior if they recognized the new EHR as useful for their job; after considering its advantages and disadvantages, it was found that the benefits of a new EHR is greater than the effort it requires to use it [3]. Since users tend to first recognize the effort required for them to adapt before recognizing the benefits of changing to a new system [3], it is essential to inform nurses of the benefits of implementing a new EHR system. Hospital leaders should communicate their rationale for implementing a new EHR system, as well as their vision for the project before or at least in the beginning of its implementation [10, 13].

Healthcare providers involved in different disciplines within a hospital collaborate and communicate with each other regarding their patients [8]; they use EHR to share patient care data and opinions regarding patient condition or care. That is, EHR resides in the center of the work of healthcare providers in the hospital; thus, colleagues’ opinions would be salient in forming the perception of users. Our finding shows that nurses tend to have more perceived usefulness and less resistance to change if their colleagues show favorable opinions toward new information systems, because they have similar working conditions. Hence, one possible strategy to accompany the implementation of a new EHR system is for hospitals to designate a champion nurse [10, 13] from each unit who should receive training in advance to guide and educate their colleagues, which could improve nurses’ opinions of the new system.

Nurses with high self-efficacy might perceive new EHR systems as easy to use, reducing their resistance to change. Hospitals could employ nurses with higher self-efficacy as champions to form favorable opinions among colleagues. Both the formal nursing informatics education and the system training are required to increase nurses’ self-efficacy [13, 42], however significant nursing schools are still required more nursing informatics education in their curriculum [43]. That is, more nursing informatics education in nursing schools is recommended to reduce user resistance behavior as well as the proper user training at implementation in a hospital.

To increase perceived ease of use, organizational support could be provided for nurses by making available adequate equipment such as up-to-date workstations as well as appropriate network bandwidth and training for nurses [13]. In addition, the process of transition towards the implementation of a new EHR system may require a larger workforce while nurses are becoming familiar with the new system. These conditions could make nurses’ transition easier and increase perceived ease of use, consequently reducing their resistance toward the new system.

This study had some limitations. First, the survey was conducted 7–19 months after participants had first encountered the new EHR system; thus, responses were reliant on participants’ memory. Consequently, this may have produced a recall bias. Second, the data was collected at a single point in time and user resistance behavior in this study was not collected as an actual behavior. Hence, this study has limitation to reveal causality on user resistance. Third, as this study was conducted in university hospitals, the findings may not be generalized to non-academic hospitals which have less resources, facilities or IT infrastructure. Nonetheless, this study is meaningful as it explores different factors to reduce user resistance against a change in the EHR system in a hospital setting, which is inevitable since hospitals will keep upgrading their systems.

## Conclusions

This study explored factors associated with user resistance behavior which were all directly or indirectly associated and provided a preliminary model of user resistance behavior to improve and understand resistance within a theoretical framework. The research model explains user resistance behavior as an outcome variable with both inhibitors and enablers drawn from user resistance and technology acceptance literature. To obtain the full benefits of EHR by mitigating user resistance behavior, it is crucial for leaders in healthcare organization carry out strategies to increase inhibitors and decrease enablers of user resistance behaviors based on the findings of this study.

## Supplementary Information

**Additional file 1.** Survey questionnaire.

## Data Availability

The datasets analyzed in this study are available from the corresponding author on reasonable request.

## Abbreviations

PV: Perceived value
CO: Colleagues’ opinion
SE: Self-efficacy for change
OS: Organizational support for change
PU: Perceived usefulness
PE: Perceived ease of use
RC: Resistance to change
UR: User resistance behavior
