# Supplementary material for: Factors associated with nurses' user resistance to change of electronic health record systems
Source: BMC Med Inform Decis Mak. 2021 Jul 17;21:218. doi: 10.1186/s12911-021-01581-z (PMC8286589; doi:10.1186/s12911-021-01581-z)
Supplement: Supplementary file 1 — Additional file 1. Survey questionnaire. [file 12911_2021_1581_MOESM1_ESM.docx]

### Additional file 1. Survey questionnaire

| Variables  (Cronbach’s alphas  in this study) | Questionnaire | Reference (Cronbach’s alphas) |
| --- | --- | --- |
| User resistance behavior  (0.748) | I did not want to change to the new way of working with the new EHR. | Kim and Kankanhalli [13]  (>0.7) |
|  | I did not cooperate in the change to the new way of working with the new EHR. |  |
|  | I was not interested in the change to the new way of working with the new EHR. |  |
|  | I opposed the change to the new way of working with the new EHR. |  |
|  | I did not change my preference for old way of working over the new way of working with the new EHR. |  |
| Resistance to change  (0.866) | I was afraid of the change to the new EHR. | Kim and Park [32] (Korean)  (0.69)  Originated from Oreg [44] |
|  | I had a bad feeling regarding the change to the new EHR. |  |
|  | The change to the new EHR made me angry. |  |
|  | The change to the new EHR stressed me out. |  |
| Perceived usefulness  (0.960) | Using the new EHR improved my job performance. | Ryu and Hwang [33] (Korean)  (0.912)  Originated from  Davis [45] |
|  | Using the new EHR enhanced my effectiveness in my job. |  |
|  | Using the new EHR made it easier to my job. |  |
|  | I found the new EHR useful in my job. |  |
| Perceived ease of use  (0.928) | Learning to operate the new EHR was easy for me. | Ryu and Hwang [33] (Korean)  (0.865)  Originated from  Davis [45] |
|  | I found it easy to get the new EHR to do what I wanted it to do. |  |
|  | My interaction with the new EHR was clear and understandable. |  |
|  | It was easy for me to become skillful at using the new EHR. |  |
|  | I found the new EHR easy to use. |  |
| Perceived value  (0.952) | Considering the time and effort that I had to spend, the change to the new way of working with the new EHR was worthwhile. | Kim and Kankanhalli [13]  (>0.7) |
|  | Considering the loss that I incurred, the change to the new way of working with the new EHR was of good value. |  |
|  | Considering the hassle that I had to experience, the change to the new way of working with the new EHR was beneficial to me. |  |
|  | Overall, changing to the new way of working with the new EHR delivered me good value. |  |
| Colleagues’ opinion  (0.802) | Almost all my colleagues thought the change to the new way of working with the new EHR was a good idea. | Kim and Kankanhalli [13]  (>0.7) |
|  | My supervisor was very supportive of the change to the new way of working with the new EHR. |  |
|  | My colleagues thought that we should all change to the new way of working with the new EHR. |  |
| Self-efficacy for change  (0.845) | There was no gap between my existing skills and knowledge and those required by the new way of working with the new EHR. | Kim and Kankanhalli [13]  (>0.7) |
|  | Based solely on my knowledge, skills and abilities, changing to the new way of working with the new EHR was easy for me. |  |
|  | I was able to change to the new way of working with the new EHR without the help of others. |  |
|  | I was able to change to the new way of working with the new EHR reasonably well on my own. |  |
| Organizational support for change  (0.919) | Specialized instruction and training concerning the change to the new way of working with the new EHR was available to me. | Kim and Kankanhalli [13]  (>0.7) |
|  | Management policies and rules facilitated the change to the new way of working with the new EHR. |  |
|  | Formal guidance was available to me in changing to the new way of working with the new EHR. |  |
|  | The management provided most of the necessary help and resources to enable me to change to the new way of working with the new EHR. |  |
|  | I was given the necessary support and assistance to change to the new way of working with the new EHR. |  |
